# Synchronous celiac artery and superior mesenteric artery compression syndrome: The effect of collateral circulation on the treatment procedure

**DOI:** 10.22088/cjim.14.2.396

**Published:** 2023

**Authors:** Ulaş Aday, Abdullah Oguz, Hikmet Özesmer, Mehmet Veysi Bahadır

**Affiliations:** 1 Department of Gastrointestinal Surgery, Dicle University School of Medicine, Diyarbakır, Turkey; 2 Department of General Surgery, Dicle University School of Medicine, Diyarbakır, Turkey

**Keywords:** Multilagament compression syndrome, Collateral circulation, Laparoscopic treatment.

## Abstract

**Background:**

Maintaining collateral circulation is highly important in the stenosis of celiac artery (CA), superior mesenteric artery (SMA), and inferior mesenteric artery (IMA). The SMA compression is commonly reported to be accompanied by the CA compression caused by the median arcuate ligament (MAL) while the synchronous compression of CA and SMA by other ligaments has been rarely reported.

**Case Presentation:**

In this report, we present a 64-year-old female patient who presented with a postprandial abdominal pain and weight loss. Initial evaluation indicated a synchronous compression of CA and SMA caused by MAL. The patient was planned for laparoscopic MAL division due to the presence of sufficient collateral circulation between the CA and SMA that was facilitated through the superior pancreaticoduodenal artery. Following laparoscopic release, the patient improved clinically and postoperative imaging indicated that the compression on the SMA was still present and the collateral circulation was sufficient.

**Conclusion:**

We suggest that laparoscopic MAL division can be the primary method of choice in cases with sufficient collateral circulation between the CA and SMA.

Mesenteric artery stenosis is encountered in only a small number of ischemia patients albeit a common entity. In the stenoses occurring in visceral arteries due to extrinsic and intrinsic reasons, wide collaterals between the celiac artery (CA), superior mesenteric artery (SMA), and inferior mesenteric artery (IMA) maintain circulation. In the presence of a 70% stenosis in CA and SMA, the clinical manifestations become pronounced and the presence of collaterals becomes more vital. Collateral circulation in patients with a stenosis of the CA and SMA is mostly facilitated through the gastroduodenal artery (GDA) (1, 2). Median arcuate ligament (MAL) syndrome describes the clinical presentation associated with direct compression of the CA by the MAL. Although the exact pathophysiology of MAL syndrome remains unknown, its clinical characteristics and treatment modalities show a wide variation. Unfortunately, not all patients respond to treatment (3). The SMA compression is commonly reported to be accompanied by the CA compression caused by MAL while the synchronous compression of CA and SMA by other ligaments has been rarely reported. The treatment to be performed in multivessel compression is relatively more complex (4-6).

In this case, we aimed to draw attention to two issues; i) CA and SMA can be compressed by separate ligaments, ii) Laparoscopic MAL division may be the first option in the presence of sufficient collateral circulation between CA and SMA.

## Case Presentation

**Figure 1:**
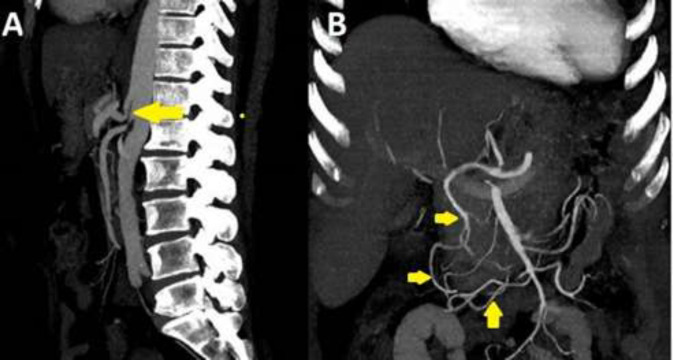
Computed tomography angiography shows the characteristic hook appearance of the proximal celiac artery and focal narrowing with poststenotic dilation. A: Significant stenosis is also observed in the superior mesenteric artery root. B: Significant collateral flow is observed between the celiac artery and the superior mesenteric artery through the gastroduodenal artery

**Figure 2:**
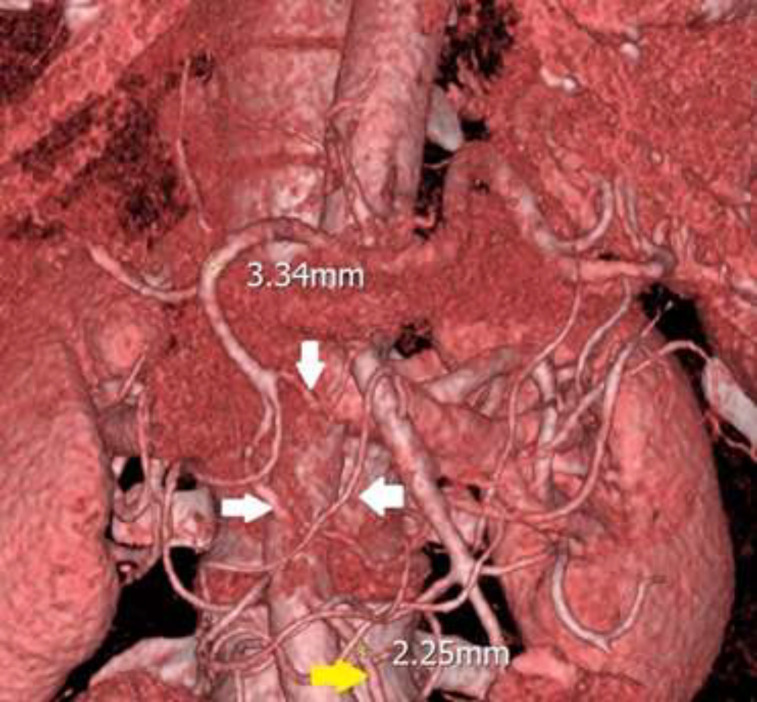
Pre-operative gastroduodenal artery (GDA) and inferior mesenteric artery diameters (yellow markers) and collaterals between GDA and superior mesenteric artery (white arrows)

**Figure 3:**
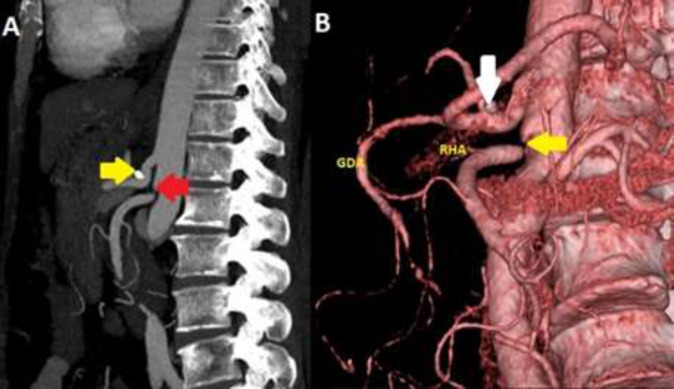
A: Postoperative computed tomography angiography shows that celiac artery compression improves, but superior mesenteric artery stenosis continues (red arrow). In addition, the clip placed in the phrenic artery is seen as hyperdense (yellow arrow). B: In the three dimensional computed tomography angiography, collateral circulation is observed between the stenotic superior mesenteric artery (yellow arrow) and the celiac artery through the gastroduodenal artery (GDA). The right hepatic artery (RHA) originates from the superior mesenteric artery. A metallic clip can be seen adjacent to the noncompressed celiac artery root (white arrow)

**Figure 4:**
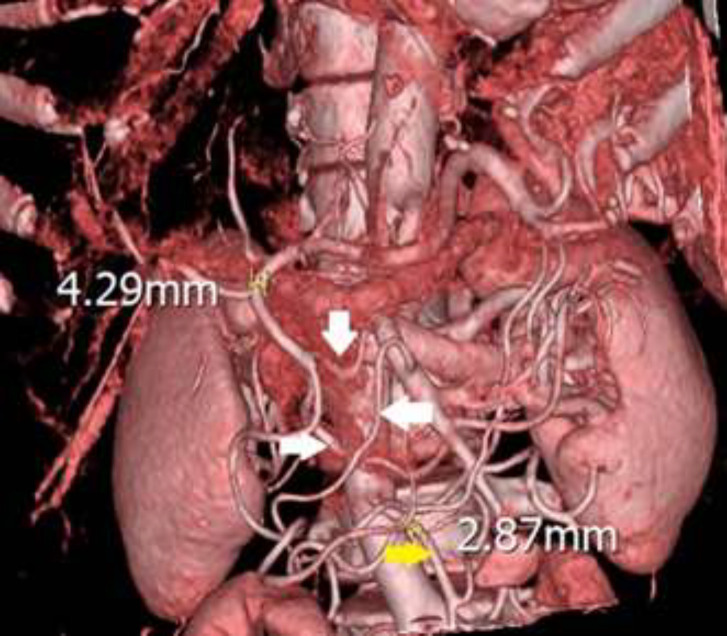
Post-operative computed tomographic angiography showing GDA and IMA diameters (yellow markers) and collaterals between GDA and SMA (white arrows)

A 64-year-old female patient presented to our clinic with the complaints of postprandial abdominal pain and weight loss. Of these, the postprandial abdominal pain was the primary presenting symptom that had been persisting for the last one year. The patient had lost 8 kg due to restricted food intake and had no history of chronic disease and drug use. Physical examination, laboratory parameters, esophagogastroduodenoscopy, colonoscopy, and abdominal ultrasonographic examination were normal. Computed tomography angiography (CTA) revealed a ~70% stenosis at the exit of the truncus coeliacus caused by the compression of MAL. Subsequently, a ~90% stenosis was detected in the proximal segment of SMA. Marked collateral circulation was observed between the CA and SMA that was facilitated through the GDA (figure 1). Colletaral was not observed between IMA and SMA (figure 2). Based on a multidisciplinary evaluation, the patient was planned for laparoscopic MAL division due to the presence of sufficient collateral circulation between the CA and SMA. The details of the surgical procedure were explained to the patient and written consent was obtained. The surgical procedure steps were performed similar to the details we have described previously (6). Left gastric artery (LGA) and arteria hepatica communis (AHC) were isolated separately and then surrounded by a vascular tape. LGA and AHC were held medially and laterally with tape and pulled to the left and caudal of the patient with the help of an endograsper. All muscle fibrous structures in the aorto-celiac axis were divided and CA was completely released. During this traction procedure, the arterial circulation of the liver was preserved by loosening the vascular tape for 5 minutes every 15 minutes. Afterwards, the celiac ganglion was excised and the procedure was completed with no complications within a total duration of 90 min. The patient was discharged uneventfully at postoperative day 5. A CTA performed at postoperative one month revealed that the CA compression disappeared completely while the compression on the SMA was still present and the collateral circulation facilitated through the GDA was sufficient (figure 3). Postoperative GDA and IMA diameter increased by 0.95 (from 3.34 mm to 4.29 mm) and 0.62 (from 2.25 mm to 2.87 mm) millimeters, respectively (figure 4). No additional treatment was recommended to the patient due to her asymptomatic clinical status. The patient is at the seventh follow-up month and she stated that she had gained 4 kg of weight throughout the follow-up period and currently has no complaints. 

## Discussion

Intra-abdominal arteries are likely to be compressed by adjacent structures. Patients with stenosis in radiological examinations are mostly asymptomatic. Treatment requires only a small part. MAL syndrome, also known as CA compression syndrome, is thought to develop with compression of the celiac artery by the MAL and/or celiac ganglion. This mechanical-anatomical compression causes an increase in compression due to the inferior displacement of the diaphragmatic crus in the inspirium. Despite being a rare entity, this syndrome leads to chronic mesenteric ischemia via external compression and is a clinical condition whose treatment practices are commonly described (3, 7). The isolated SMA compression requiring treatment has been rarely reported in the literature, mainly in case reports investigating synchronous CA and SMA compression. In those case reports, however, the stenosis was shown to be caused only by MAL (5). Patients with multiple vascular stenosis may remain asymptomatic for years, which can be explained by the well-functioning collateral vascular pathways of the mesenteric circulation. The most common collateral pathways found between the CA and the SMA are the pancreaticoduodenal arcades and occasionally the arc of Buhler. Similarly collaterals are formed between IMA and SMA. Common connections between the SMA and IMA include the marginal artery of Drummond and the arc of Riolan, and the patency of this collateral network is very important in patients with combined CA and SMA stenosis because circulation continues in this way. However, if there is not enough collateral between IMA and SMA, it becomes symptomatic (2, 4). On the other hand, the coexistence of CA and SMA stenosis requiring treatment is also extremely rare. van Petersen et al. (1) detected a >70% stenosis in both CA and SMA in 21 (9%) out of 228 patients on mesenteric angiography. In these patients, the collateral circulation was facilitated through the GDA in three patients and through the arcade of Riolan or Drummond in 15 patients. Synchronous SMA compression was not detected in a different study of the same author, which included 129 patients treated for MAL syndrome. The authors suggested that patients with MAL syndrome and with extensive collateral circulation benefited less from the treatment compared to patients without this type of collateral circulation (2). Arazińska et al. (4) evaluated 103 patients with MAL syndrome and detected the coexistence of MAL syndrome with CA and SMA stenosis in four patients and detected collateral circulation in 23 patients. To our knowledge, there is little or no documentation of the synchronous compression of CA and SMA caused by different ligaments in the literature. This paucity could be associated with the avoidance of additional diagnostic procedures following MAL division due to the clinical improvement enabled by collateral circulation. Accordingly, future studies are needed to substantiate the available data on this topic. In the present study, the SMA stenosis was considered to be associated with MAL during the initial diagnosis and the compression on SMA was found to be continuing in postoperative imaging. Based on these findings, we considered that the stenosis was induced by another ligament or by a congenitally atresic segment of those ligaments. Nevertheless, there is no definitive data regarding this phenomenon since SMA cannot be evaluated by open surgery (6-8). 

Open surgical techniques are now being replaced by less invasive laparoscopic techniques. Anterior transperitoneal laparoscopic division of MAL is safely administered in numerous centers around the world (3, 7-9). The laparoscopic procedure may be converted to an open procedure when the dissection site cannot be visualized sufficiently and the bleeding cannot be controlled. Nonetheless, there is no standard and common approach for the visualization of the aorto-celiac axis. Even so, encircling the AHC and LGA with soft vessel tape and performing their traction of the arteries in the caudal and left direction allows optimal visualization as well as a safe surgical procedure. Additionally, this procedure can be completed with no need for esophageal suspension (6). If symptoms do not resolve with a persistent stenosis on the post-operative CT scan after MAL release, selective revascularization with endovascular stenting can be performed (4). In this case, angioplasty for SMA was not performed due to asymptomatic clinical conditions. Synchronous compression of the CA and SMA is extremely rare and remains to be elucidated. Laparoscopic MAL division alone can be the primary method of choice in cases with sufficient collateral circulation between the CA and SMA.
